# Inhibitory Effect of Tanshinone IIA on Rat Hepatic Stellate Cells

**DOI:** 10.1371/journal.pone.0103229

**Published:** 2014-07-30

**Authors:** Ya-Wei Liu, Yi-Tsau Huang

**Affiliations:** 1 Institute of Traditional Medicine, School of Medicine, National Yang-Ming University, Taipei, Taiwan; 2 National Research Institute of Chinese Medicine, Ministry of Health and Welfare, Taipei, Taiwan; Harvard Medical School, United States of America

## Abstract

**Background:**

Anti-inflammation via inhibition of NF-κB pathways in hepatic stellate cells (HSCs) is one therapeutic approach to hepatic fibrosis. Tanshinone IIA (C_19_H_18_O_3_, Tan IIA) is a lipophilic diterpene isolated from *Salvia miltiorrhiza* Bunge, with reported anti-inflammatory activity. We tested whether Tan IIA could inhibit HSC activation.

**Materials and Methods:**

The cell line of rat hepatic stellate cells (HSC-T6) was stimulated with lipopolysaccharide (LPS) (100 ng/ml). Cytotoxicity was assessed by MTT assay. HSC-T6 cells were pretreated with Tan IIA (1, 3 and 10 µM), then induced by LPS (100 ng/ml). NF-κB activity was evaluated by the luciferase reporter gene assay. Western blotting analysis was performed to measure NF-κB-p65, and phosphorylations of MAPKs (ERK, JNK, p38). Cell chemotaxis was assessed by both wound-healing assay and trans-well invasion assay. Quantitative real-time PCR was used to detect gene expression in HSC-T6 cells.

**Results:**

All concentrations of drugs showed no cytotoxicity against HSC-T6 cells**.** LPS stimulated NF-κB luciferase activities, nuclear translocation of NF-κB-p65, and phosphorylations of ERK, JNK and p38, all of which were suppressed by Tan IIA. In addition, Tan IIA significantly inhibited LPS-induced HSCs chemotaxis, in both wound-healing and trans-well invasion assays. Moreover, Tan IIA attenuated LPS-induced mRNA expressions of *CCL2, CCL3, CCL5, IL-1β, TNF-α, IL-6, ICAM-1, iNOS,* and *α-SMA* in HSC-T6 cells.

**Conclusion:**

Our results demonstrated that Tan IIA decreased LPS-induced HSC activation.

## Introduction

Liver fibrosis is a wound-healing, scarring response to such chronic injuries as hepatitis B and C, alcoholic liver disease, nonalcoholic steatohepatitis, and autoimmune hepatitis [1], [2]. Hepatic fibrosis has been recognized as a dynamic and at some stages, reversible process [3]. In the normal liver, hepatic stellate cells (HSCs) are myofibroblasts located in the space of Disse. Quiescent HSCs are primarily responsible for storing huge amounts of vitamin A in lipid droplets [4]-[6]. Activation of HSCs is the contributing cause of liver fibrogenesis and characterized by phenotypic transformation with diverse functional changes, including proliferation, contractility, cytokine secretion, chemotaxis, fibrogenesis, and matrix degradation [6]. Namely, HSCs are induced to trans-differentiate from quiescent into activated form by inflammatory mediators or growth factors [1], [5], [7].

Lipopolysaccharide (LPS, or endotoxin) is a major component of the outer membrane of Gram-negative bacteria. It has been reported that LPS levels in patients with cirrhosis are increased in both systemic and portal venous blood [8], [9]. LPS is the ligand of the pattern recognition receptor, Toll-like receptor 4 (TLR4), and powerfully stimulates host innate immune responses [8], [9]. LPS instigates the consequent signals downstream and leads to the activation of the transcription factors NF-κB and AP-1, resulting in the induction of potent innate immune responses [10], [11]. Scientific reports mentioned that myofibroblasts are also critical innate immune sensors induced by LPS and can themselves generate various inflammatory effectors including chemokines, cytokines, and oxygen radicals [12]. LPS triggers high levels of TLR4 expression in HSCs [13], [14]. In both *in vitro* and *in vivo* studies, gut derived LPS is an important activator for Kupffer cells and HSCs in liver injury [14]–[16].

*Salvia miltiorrhiza Bae.* (*S. miltiorrhiza*), the root of *Labiatae* plant, has been used to treat heart, metabolic diseases and hepatitis for a long time in Chinese medicine [17]. Several reports including ours have shown that *S. miltiorrhiza* extracts exert both *in vitro* and *in vivo* inhibitory effects against various models of hepatic fibrosis [18]–[20]. Salvianolic acid B (C_36_H_30_O_16_, Sal B) is a major component in aqueous hydrophilic extract of *S. miltiorrhiza*. Sal B has been demonstrated to inhibit the activation of ERK and NF-κB in TNF-α-stimulated endothelial cells [21]. Previous studies indicated that *S. miltiorrhiza* extracts inhibited carbon tetrachloride (CCl_4_)-induced P450 2E1 and iNOS protein in rats. Furthermore, Sal B suppressed CCl_4_-stimulated accumulation of reactive oxygen species (ROS) in hepatocytes and HSCs, and also HSC activation [22], [23]. Tanshinone IIA (C_19_H_18_O_3_, Tan IIA) is one of the main lipophilic compounds of *S. miltiorrhiza.* In an *in vitro* study, Tan IIA has been revealed to suppress production of NO, expressions of iNOS and IL-1β in activated RAW 264.7 cells [24]. An *in vivo* study showed that Tan IIA induces activation of the cytochrome P450 mixed-function oxidase system in C57B/6J mice [25], [26].

There is still no clinically efficacious drug specifically against liver fibrosis [2]. The potential of Tan IIA as an agent against liver fibrosis is not clarified. The present study was undertaken to explore the potential effect of Tan IIA on LPS-induced HSCs, in comparison with Sal B.

## Materials and Methods

### Cell culture and treatment

The HSC-T6, a generous gift from Prof. S.L. Friedman, is an immortalized cell line of rat HSCs [4]. HSC-T6 cells were incubated in Waymouth MB 752/1 Medium (Sigma-Aldrich, St. Louis, MO, USA) containing 10% fetal bovine serum (FBS, pH 7.0; Gibco BRL, Gaithersburg, MD, USA) at 37°C in 5% CO_2._

### Preparation of drugs

Tan IIA was obtained from Sigma-Aldrich, and Sal B was isolated from the roots of *S. miltiorrhiza*, as previously reported [23]. For *in vitro* experiments, Tan IIA and Sal B were dissolved in dimethyl sulfoxide (DMSO, Sigma-Aldrich) and diluted with medium to give a DMSO concentration below 0.1%. LPS (100 ng/ml, Sigma-Aldrich) was used in all experiments for stimulation.

### Cell viability assay

3-(4,5-dimethylthiazol-2-yl)-2,5-diphenyltetrazolium bromide (MTT) assay was performed to exclude the cytotoxicity of drugs to HSCs. Cells (7.5×10^4^ cells/well) were seeded in 24-well plates with FBS-free medium for 24 hr, and with Tan IIA (1, 3 and 10 µM) and Sal B (200 µM) for another 24 hr at 37°C. After incubation with drugs, MTT assay was used to evaluate the cell viability of HSC-T6 cells. The cells were incubated with minimum essential medium containing 0.1 mg/ml MTT (Sigma-Aldrich) for 30 min. The formazan particle was dissolved with DMSO and measured using an enzyme-linked immunosorbent assay reader, according to the method of our previous report [27].

### NF-κB responsive luciferase assay

Cells (7.5×10^4^ cells/well) were seeded in 24-well plates with FBS-free medium at 37°C the day before transfection. Transfection was performed with transfection reagent Fugene-6 (Roche, Basel, Switzerland) according to manufacturer's specifications. NFκB-Luc reporter construct (1 µg/well) (Stratagene, La Jolla, CA, USA) was added to cells along with plasmid CMV-β-galactosidase (CMV-β-gal, 0.2 µg/well; Promega, Madison, WI, USA) and transfection reagent (15 µl/24-well plate). CMV-β-gal served as an internal control to normalize the transfection efficiency. After 24 hr incubation, cell cultures were treated with Tan IIA (1, 3 and 10 µM) or Sal B (200 µM) for 30 min. LPS was then added to stimulate NF-κB activity for 6 hr. Cell lysates were harvested with diluted reporter lysis 5X buffer (Promega). 24-well plates were added reporter lysis buffer (100 µl/well), and 20 µl cell lysate mixed with 100 µl luciferin in white 96-well plates. Luminescence was detected by luminometer-VICTOR2 Multilabel Counter (Perkin Elmer Inc., Waltham, MA, USA), as previously reported by us [27], [28].

### Western blotting analysis

HSC-T6 cells were seeded on 6 well-plates (1×10^6^ cells/well). After culturing for 24 hr, serum-starved cells were pretreated with Tan IIA (10 µM) or Sal B (200 µM) for 1 hr, then exposed to LPS for 15 min for harvesting NF-κB-p65 and MAPKs (ERK, JNK and p38), and exposed to LPS for 24 hr for harvesting α-SMA. Nuclear extracts of HSCs for NFκB-p65 translocation were prepared according to our previous study [29]. Whole cell protein extracts for measurement of ERK, JNK, p38 and α-SMA proteins were harvested with RIPA buffer. Cell lysates were separated with 10% SDS-PAGE (30 µg/lane; Pierce, Rockford, IL, USA) and transferred onto Immobilon-PVDF (Millipore, Billerica, MA, USA). The following antibodies have been used in various dilutions: anti-NFκB-p65 (1∶5000, Santa Cruz Biochnology, Santa Cruz, California, CA, USA), anti-PCNA (1∶10000, Cell Signaling Technology, Danvers, MA, USA), anti-phospho-ERK (1∶5000), anti-phospho-JNK (1∶5000), anti-phospho-p38 (1∶5000, Cell Signaling Technology, Danvers, MA, USA), anti-α-SMA (1∶10000, Calbiochem-Novabiochem, San Diego, CA, USA), and anti-α-tubulin (1∶10000, Santa Cruz Biochnology, Santa Cruz, California, CA, USA). The specific horseradish peroxidase conjugated secondary antibodies (1∶10000, Cell Signaling Technology, Danvers, MA, USA) were used and blots were developed using enhanced chemiluminescence detection reagent and visualized on digital detection imaging system.

### Immunofluorescent staining

HSC-T6 cells were seeded on glass cover slips in 6 well-plates (1×10^6^ cells/well) with medium at 37°C for 1 day incubation. Serum-starved cells were pretreated with Tan IIA (10 µM) or Sal B (200 µM) for 1 hr, then exposed to LPS for 15 min. The cells were fixed, permeabilized, and blocked. Samples were incubated with an antibody against NFκB-p65 (1∶500, Santa Cruz Biochnology, Santa Cruz, CA, USA) incubated in 4°C overnight, and then mounted with 4′,6-diamidino-2-phenylindole dihydrochloride (DAPI, Sigma-Aldrich). The cells were visualized on LSM780 confocal microscope (Zeiss, Oberkochen, Germany) using a digital imaging system. The scale bar was added using Metamorph software.

### Wound-healing assay

HSC-T6 cells were seeded on collagen coated 24-well plates [28], [30], [31] at a density of 9×10^4^ cells/well. After 24 hr serum starvation, a monolayer of cells was scratched by a 200 µl tip in serum-free medium. Cells were pre-treated with Tan IIA (10 µM) and Sal B (200 µM) for 1 hr, then incubated in the presence of LPS for 24 hr. HSC-T6 cell migration was quantified by the area of migrated cells to the scratched cell-free zone after 24 hr, and measured by Image-Prop PLUS software.

### Trans-well invasion assay

Serum-starved HSC-T6 cells (1×10^5^ cells/trans-well) were cultured on 24-well chemotaxis chamber Millicell (Merck Millipore, Darmstadt, Germany) with 8 µM pores, which were pre-coated Matrigel Matrix (100 µl/trans-well; BD Biosciences, San Jose, CA, USA) for 1 hr at room temperature, and then washed with serum-free medium. The bottom wells were filled with serum-free medium co-treating with LPS plus Tan IIA (10 µM) or Sal B (200 µM) for 24 hr, and the entire chamber was incubated at 37°C for 24 hr. Cells migrated to the lower surface of the membrane were stained with hematoxylin (Sigma-Aldrich) to define the cell nuclei, as previously reported by us [32].

### Quantitative real-time polymerase chain reaction analysis

HSCs (1×10^6^ cells/well) were seeded on 6 well-plates. After culturing for 24 hr, serum-starved cells were pretreated with Tan IIA (10 µM) or Sal B (200 µM) for 1 hr, then exposed to LPS for 12 hr. Total RNA was extracted from HSC-T6 cells using the TRIzol Reagent (Life technologies, Carlsbad, CA, USA). RNA was prepared by reverse transcription using oligo-dT and dNTP, and each sample was added with RT kit, Life technologies). Quantitative real-time PCR was performed by the StepOnePlus Quantitative Real-Time PCR System (Life technologies), according to the manufacturer's instructions. The sequences of primers for quantitative real-time PCR are listed in Table S1.

### Statistical analysis

Data were expressed as mean ± S.E.M. Statistical analysis was obtained using one-way ANOVA. A *p* value less than 0.05 was considered as significantly different.

## Results

### Tanshinone IIA has no cytotoxicity in HSC-T6 cells

**Figure 1:**
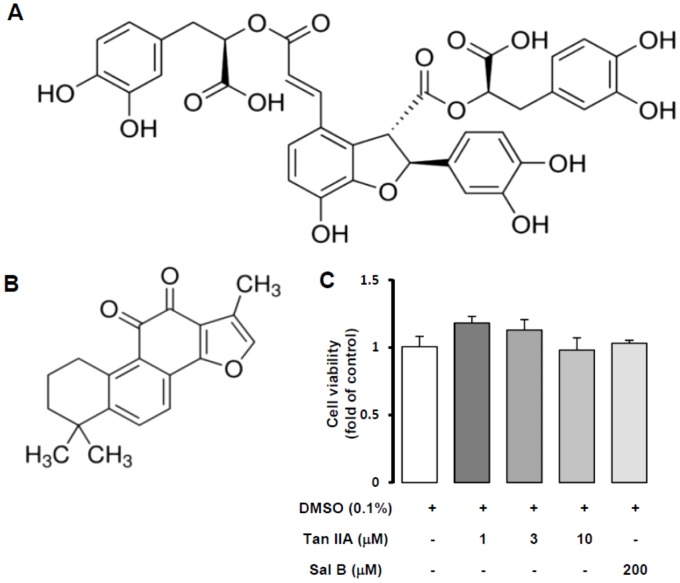
Chemical structures and cytotoxicity of salvianolic acid B and tanshinone IIA. Chemical structures of Sal B (A) and Tan IIA (B). (C) MTT assay was performed to assess HSC-T6 cells viability with Tan IIA (1, 3, and 10 µM) and Sal B (200 µM) treatment for 24 hr. There was no cytotoxicity in all concentrations of drugs. n = 3.

Both Sal B and Tan IIA (Figure 1A and B) are key constituents of *S. miltiorrhiza*, the former coming from aqueous extract and the latter being in lipophilic part. According to our previous studies, Sal B has already been found to protect hepatic function, such as inhibiting CCl_4_ induced ROS accumulation in hepatocytes and HSC activation [21], [23]. Therefore, we further studied bioactivities of Tan IIA against HSC activation. Using MTT assays, neither Tan IIA (1, 3 and 10 µM) nor Sal B (200 µM) showed cytotoxicity to HSC-T6 cells for 24 hr (Figure 1C). To confirm whether there was proliferative effect of Tan IIA and Sal B in HSC-T6 cells, we used immunofluorescent staining of Ki67. The results show that there was no increase in proliferation by Tan IIA or Sal B (Figure S1).

### Tanshinone IIA attenuated LPS-induced NF-κB activation in HSC-T6 cells

**Figure 2:**
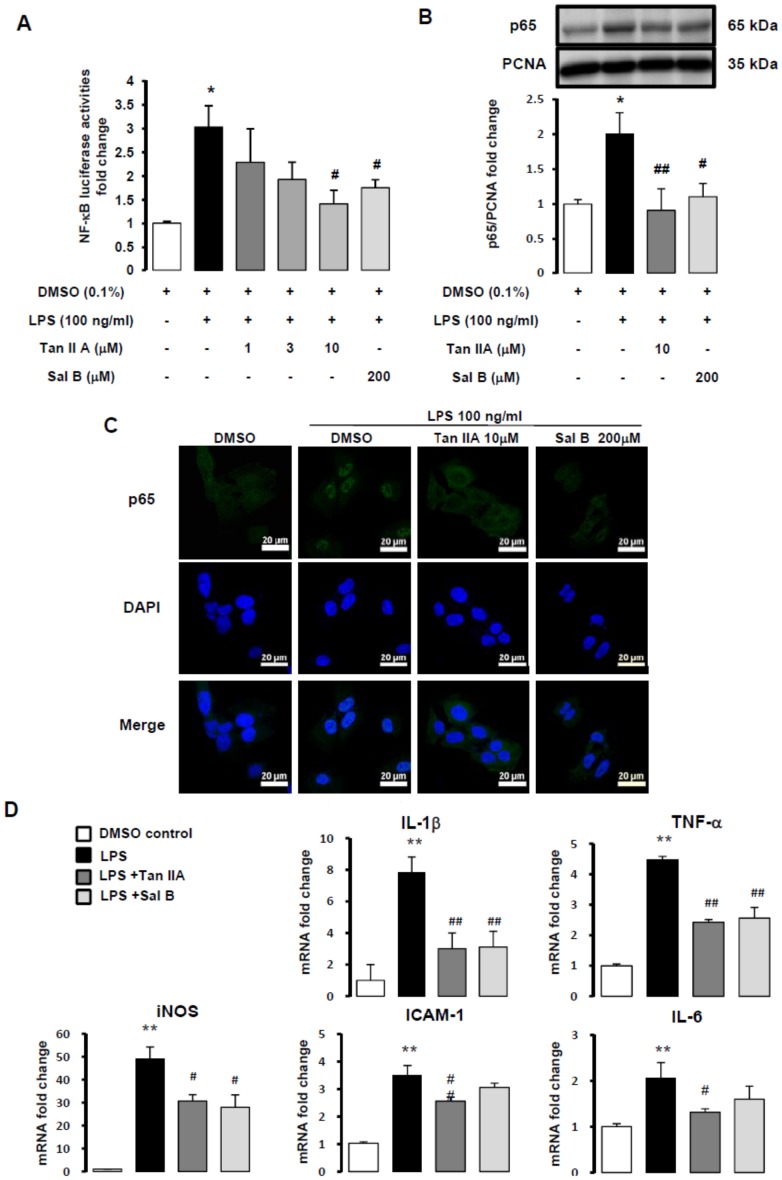
Tan IIA decreased LPS-induced NF-κB activity in HSC-T6 cells. (A) NF-κB activity of HSC-T6 cells pre-transfected with NF-κB-Luc plasmid for 24 hr. Cells were pretreated with Tan IIA (1, 3, 10 µM) and Sal B (200 µM) for 30 min, after 6 hr LPS (100 ng/ml) stimulation, NF-κB activity was detected by luminescence. CMV-βgal was used as internal control to normalize the transfection efficiency. *, *p*<0.05 versus DMSO control; ^#^, *p*<0.05 compared with LPS-induced only group; n = 3. (B) The nuclear translocation of NF-κB-p65 was assessed by Western blotting assay. PCNA expression served as a loading control in nuclear protein. The nuclear translocation of NF-κB-p65 was pre-treated with Tan IIA (10 µM) and Sal B (200 µM) for 1 hr, then stimulated with LPS (100 ng/ml) for 15 min. *, *p*<0.05 versus DMSO control; ^#^, *p*<0.05 versus LPS-induced only group; ^##^, *p*<0.01 versus LPS-induced only group; n = 5. (C) HSC-T6 cells were pre-treated with Tan IIA (10 µM) and Sal B (200 µM) for 1 hr, then stimulated with LPS (100 ng/ml) for 15 min. NF-κB-p65 (green) and DAPI (pseudo-red, original color being blue) were detected by immunofluorescent staining assay, and white arrows showed NF-κB-p65 colocalized with nuclei. (D) Quantitative real-time PCR analysis of HSC-T6 cells pretreated with Tan IIA (10 µM) and Sal B (200 µM) for 1 hr. After LPS (100 ng/ml) exposure, total RNA of HSC-T6 cells were extracted for Quantitative real-time PCR assays. The RNA expressions of *IL-1β, TNF-α, IL-6, ICAM-1* and *iNOS* were detected. **, *p*<0.01 versus DMSO control; ^#^, *p*<0.05 and ^##^, *p*<0.01 compared with LPS-induced only group; n = 3-5.

NF-κB is a transcription factor crucially involved in inflammatory responses. We used a reporter gene assay to detect whether LPS-induced NF-κB activities were downregulated by Tan IIA. Cells were pretreated with Tan IIA (1, 3 or 10 µM) or Sal B (200 µM) for 30 min, and stimulated with LPS (100 ng/ml) for 6 hr. LPS-induced NF-κB luciferase activities were significantly suppressed by both Tan IIA (10 µM) and Sal B (200 µM, Figure 2A). We used NF-κB inhibitor- pyrrolidine dithiocarbamate (PDTC) for comparison in the Luciferase assay. Furthermore, PDTC also effectively downregulated LPS-stimulated mRNA expressions of *IL-1β, TNF-α, iNOS, ICAM-1* and *IL-6* genes (Figure S2). The NF-κB heterodimer (p50/p65) is sequestered in the cytoplasm by IκB-α in the un-stimulated normal cells. If cells are stimulated with diverse agents, p50/p65 is released from IκB-α. NF-κB activity is induced following p65 translocation into nucleus from cytoplasm. In Western blotting analysis, we found that nuclei contents of NF-κB-p65 subcomponent was increased by LPS (100 ng/ml), which was attenuated by both Tan IIA and Sal B significantly (Figure 2B). In addition, with immunofluorescent imaging, we also observed that LPS-triggered NF-κB-p65 nuclei translocation from the cytoplasm was ameliorated by Tan IIA and Sal B (Figure 2C). NF-κB-p65 is green and DAPI represents the nuclei part which was pseudo-colored from blue to red by Metamorph software. When NF-κB-p65 translocation was induced by LPS, merged images indicated colocalization of NF-κB- p65 with nuclei showing orange color (bar, 100 µm).

We used gene transcription assays to test our hypothesis that inflammatory signaling is affected by Tan IIA and Sal B *in vitro*. Quantitative real-time PCR analysis of whole cell RNA demonstrated that Tan IIA suppressed mRNA expressions of *IL-1β, TNF-α, iNOS, ICAM-1, and IL-6* genes in LPS-stimulated HSC-T6 cells, however, there was no significance in *ICAM-1 and IL-6* genes in LPS-stimulated HSC-T6 cells with Sal B treatment (Figure 2D, gene subsequences of forward and reverse primers are described in Table S1). LPS-induced *IL-1β* reaches to 7.8 folds compared with control, and *iNOS* is dramatically to 49.2 folds. Results suggested that LPS had great ability to trigger iNOS production in HSCs. Of note, Tan IIA exerted better and significant inhibition of LPS-induced mRNA expressions of *ICAM-1* and *IL-6* in HSC-T6 cells.

Taken together, our results suggest that both Tan IIA and Sal B might have inhibitory effect in LPS-mediated NF-κB signaling by decreasing NF-κB luciferase activities and NF-κB-p65 nuclear translocation. Otherwise, there was distinct inhibition of Tan IIA and Sal B in related genes expression of inflammatory signaling.

### LPS-stimulated phosphorylations of mitogen-activated protein kinases (MAPKs) were inhibited by Tan IIA in HSC-T6 cells

**Figure 3:**
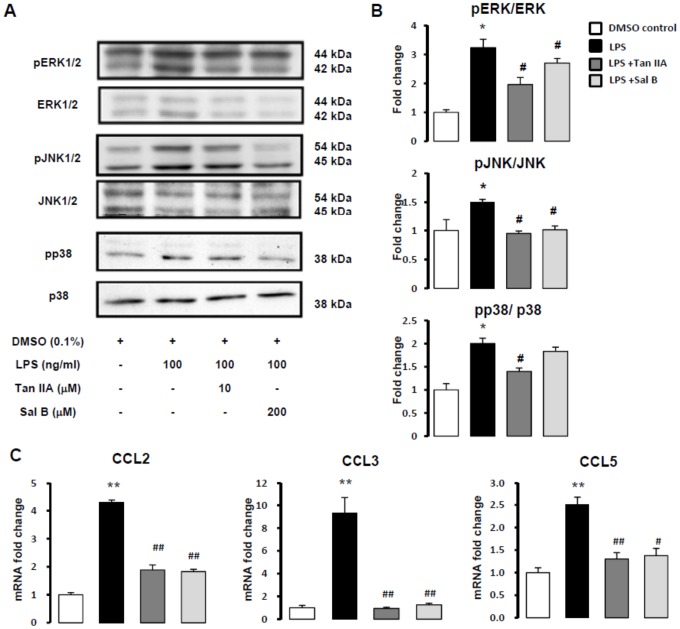
Tan IIA attenuated LPS-stimulated MAPKs (pERK1/2, pJNK1/2 and p38) signaling. (A) Phosphorylations of MAPKs were detected by Western blotting assays in HSC-T6 cells pretreated with Tan IIA (10 µM) and Sal B (200 µM) for 1 hr, and stimulated by LPS (100 ng/ml) for 15 min. (B) The amounts of phosphorylations of pERK1/2, total ERK1/2, pJNK1/2, total JNK1/2, p38 and total p38 were measured by Western blotting assay. *, *p*<0.05 versus DMSO control; ^#^, *p*<0.05 versus LPS-induced only group; n = 5. (C) Quantitative real-time PCR analysis of HSC-T6 cells pretreated with Tan IIA (10 µM) and Sal B (200 µM) for 1 hr. After LPS (100 ng/ml) exposure, total RNA of HSC-T6 cells were extracted for Quantitative real-time PCR assays. The RNA expressions of *CCL2, CCL3* and *CCL5* were detected. **, *p*<0.01 versus DMSO control; ^#^, *p*<0.05 and ^##^, *p*<0.01 compared with LPS-induced only group; n = 3-5.

Mitogen-activated protein kinases (MAPKs) are involved in key cellular functions, such as proliferation, inflammatory responses, differentiation and migration. MAPKs include ERK, JNK and p38 [33]. LPS-induced phophorylations of ERK, JNK and p38 were mostly attenuated by Tan IIA (10 µM) and Sal B (200 µM), except that Sal B had a slight but non-significant tendency of inhibiting p38 (Figure 3A and B). To sum up, Tan IIA was superior to Sal B in inhibiting LPS-stimulated phosphorylations of MAPKs in HSC-T6 cells. Reports showed that several chemokines are crucially associated with MAPKs pathway, such as chemokine (C-C motif) ligands-CCL2, CCL3 and CCL5 [34]-[36]. Therefore, we used quantitative real-time PCR to assess whether Tan IIA and Sal B showed the ability in downregulating LPS-induced CCL2, CCL3, and CCL5. It has been observed that Tan IIA and Sal B abolished LPS-induced mRNA expressions of *CCL2, CCL3* and *CCL5* (Figure 3C). Remarkably, LPS triggered gene expression of *CCL3* to 9.3 times (9.30 ±1.38), and Tan IIA and Sal B inhibited *CCL3* efficiently.

### Tan IIA suppressed LPS-stimulated chemotaxis of HSC-T6 cells

**Figure 4:**
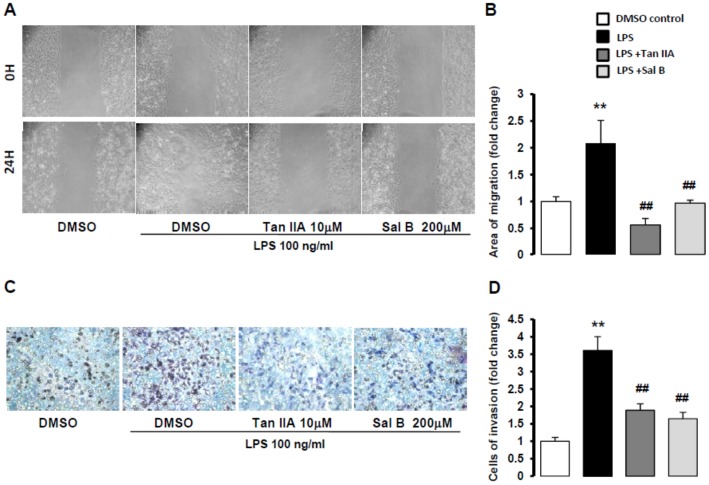
Tan IIA inhibited LPS-induced HSC-T6 migration. (A) Cell migration of HSC-T6 cells cultured in collagen-coated 24-well plates. After 24 hr, cells were scratched with a 200 µl tip to take away a monolayer. Tan IIA (10 µM) and Sal B (200 µM) pretreated HSC-T6 cells for 1 hr, and then LPS (100 ng/ml) stimulated cells to migrate to cell-free area. (B) Quantification of wound-healing assay by counting the area of cell migration. **, *p*<0.01 versus DMSO control; ^##^, *p*<0.01 compared with LPS-induced only group; n = 4. (C) HSC-T6 cells cultured on the upper chamber of trans-well for 24 hr that were treated by LPS (100 ng/ml), Tan IIA (10 µM) and Sal B (200 µM) in the lower chamber. HSC-T6 cells crawled to the other side of trans-well membrane via the pores (8 µm). (D) Quantification of trans-well assay by counting the number of cells invasion. **, *p*<0.01 versus DMSO control; ^##^, *p*<0.01 compared with LPS-induced only group; n = 3.

Chemokines secretion is required to cell migration [35], [37], and elevated HSC motility is crucially involved in hepatic fibrogenesis. On the basis of Tan IIA and Sal B against LPS-induced *CCL2, CCL3* and *CCL5* mRNA, which are key chemokines associated with chemotaxis. We determined to use wound-healing assay and trans-well invasion assay to exam Tan IIA and Sal B on LPS-induced HSC motility. Wound-healing assay revealed that with 24 hr exposure to LPS, HSC-T6 cells showed marked migration to the cell-free zone, which was attenuated by both Tan IIA and Sal B (Figure 4A and B). The trans-well invasion assay also showed that both Tan IIA and Sal B reduce 24 hr LPS-stimulated migration of HSC-T6 cells (Figure 4C and D). Both assays demonstrated Tan IIA and Sal B had great ability to suppress LPS-induced HSC migration.

### Tan IIA inhibited LPS-induced fibrogenic α-SMA production in HSC-T6 cells

**Figure 5:**
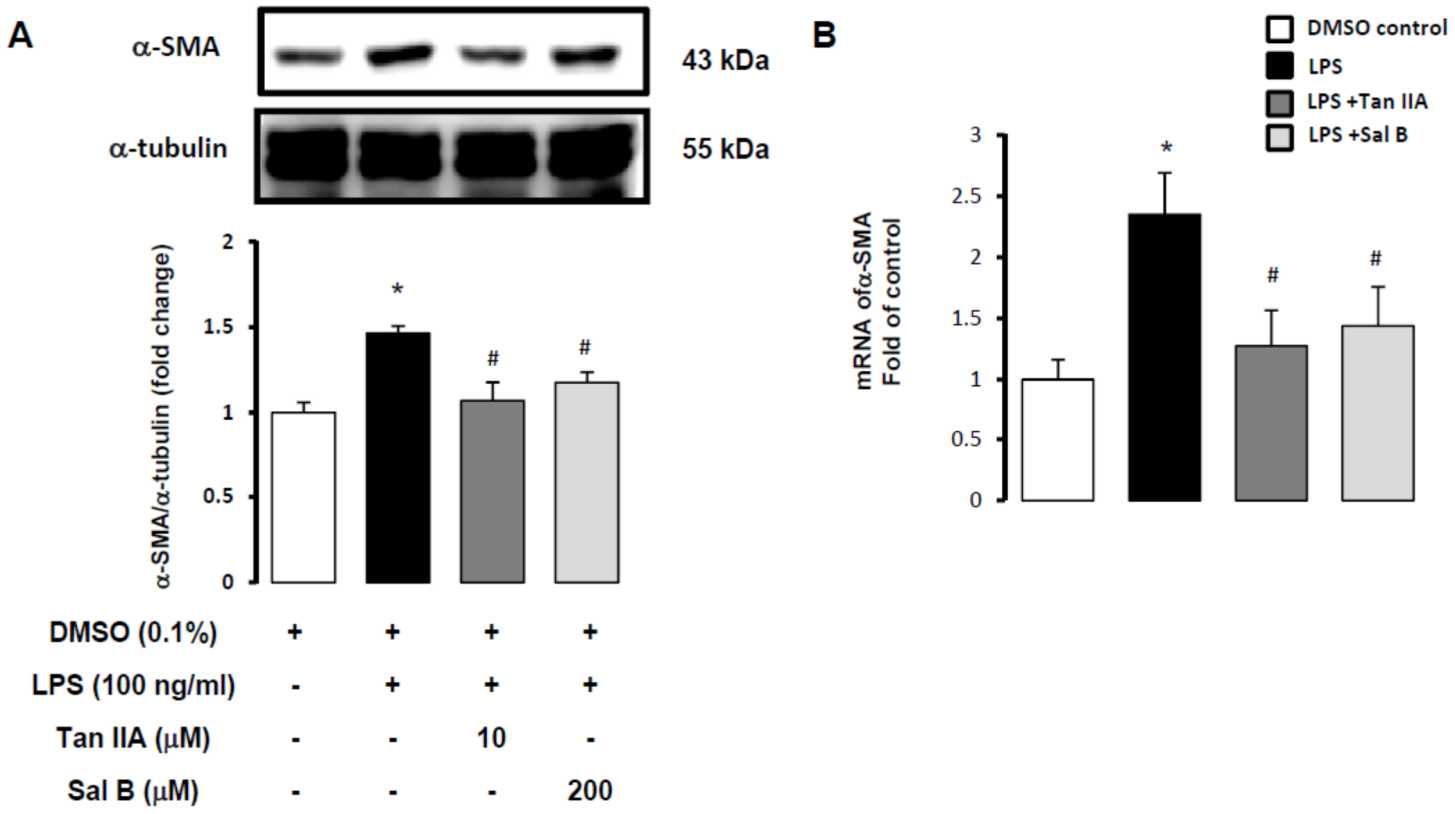
Tan IIA inhibited LPS-induced α-SMA production in HSC-T6. (A) α-SMA was detected by Western blotting assay in HSC-T6 cells pretreated with Tan IIA (10 µM) and Sal B (200 µM) for 1 hr, and stimulated by LPS (100 ng/ml) for 1 hr. α-tubulin expression served as a loading control. *, *p*<0.05 versus DMSO control; ^#^, *p*<0.05 versus LPS-induced only group; ^##^, *p*<0.01 versus LPS-induced only group; n = 4. (B) Quantitative real-time PCR analysis of HSC-T6 cells pretreated with Tan IIA (10 µM) and Sal B (200 µM) for 1 hr. After LPS (100 ng/ml) exposure, total RNA of HSC-T6 cells were extracted for Quantitative real-time PCR assays. The RNA expression of *α-SMA* was detected. *, *p*<0.05 versus DMSO control; ^#^, *p*<0.05 compared with LPS-induced only group; n = 5.

HSC activation is the major change of liver fibrosis. α-SMA production and accumulation are main characteristics in HSC activation. We observed clear chemotaxis of LPS-induced HSC, and results showed that Tan IIA and Sal B both inhibited cell mobility caused by LPS. We determined to use Western blotting analysis to assess the change of α-SMA in LPS-stimulated HSC. Results revealed that Tan IIA and Sal B downregulated LPS-induced α-SMA protein production (Figure 5A), and this is supported by gene transcription assays that showed Tan IIA and Sal B reduced mRNA of *α-SMA* (Figure 5B). Our previous reports also indicated Sal B could inhibited TGF-β and CCl_4_-induced HSC activation and our present data give more information about ability of Sal B in inhibiting LPS-induced HSC activation. Moreover, our results demonstrated that the lipophilic compound Tan IIA showed more powerful inhibition in α-SMA production than the hydrophilic compound Sal B.

## Discussion

In the present study, we have observed that Tan IIA and Sal B, two different compounds of *S. miltiorrhiza* could decrease LPS-stimulated HSC activation by reducing NF-κB luciferase activity, NF-κB-p65 translocation, phosphorylations of MAPKs, chemotaxis of HSCs, and mRNA expressions of several pro-inflammatory and pro-fibrotic genes. There was no cytotoxicity to HSC-T6 cells at the working concentrations of both Tan IIA (1, 3 and 10 µM) and Sal B (200 µM).

Mammalian cells recognize the presence of pathogen associated molecular patterns (PAMPs) through a group of receptor complexes [38]. Originally, TLRs were identified as mammalian homologues of *Drosophila* Toll, and the function is pattern recognizing [39], [40]. TLRs crucially initiate innate immunity and activate downstream intracellular signalling pathways resulting in an induction of innate immune response. LPS is the most important ligand of TLRs, and TLRs are expressed on various kinds of cells in the liver, including Kupffer cells, hepatocytes and HSCs [38]. Ligands binding to TLRs activate MyD88-dependent and -independent signalling pathways. The MyD88-dependent pathway is related to NF-κB translocation into nuclei, and activation of MAPKs and proinflammatory cytokines [10], [41]. Activation of TLRs in HSCs by LPS has been reported to be involved in various acute and chronic liver diseases. Our present results demonstrated that LPS efficiently induced the NF-κB signaling pathway, phosphorylations of MAPKs, and several gene transcriptions related to inflammation and fibrogenesis. In addition, LPS stimulated HSC chemotaxis notably.

*S. miltiorrhiza* has been prescribed for a variety of maladies in Chinese medicine [42]. Several studies including ours have demonstrated inhibitory effects of *S. miltiorrhiza* extracts against hepatic fibrosis [17], [18], [43]. Tan IIA and Sal B are lipophilic and hydrophilic compounds of *S. miltiorrhiza*, respectively. In the literature, Tan IIA has been shown to inhibit LPS-induced NF-κB mobilization and ERK phosphorylation in rat macrophages [44]. Tan IIA has also been reported to ameliorate ischemia/reperfusion injury, with inhibition of NF-κB and MAPKs signaling pathways [45]. Moreover, there had been evidence that Tan IIA protected mice from immune-mediated liver injury via NF-κB and IFN-γ/STAT1 signaling pathways [46]. Additionally, Tan IIA has also been reported to suppress LPS- and ethanol-induced Kupffer cell sensitization, and inhibit formation of reactive oxygen/nitrogen species [47]. Sal B has been shown to diminish LPS-inducible TNF-α production in macrophages [48]. Our present study illustrated that LPS-induced NF-κB activation and phophorylations of MAPKs were reduced by Tan IIA and Sal B in another cell type, HSCs. Cell migration is a characteristic of HSC activation. A previous report demonstrated that Tan IIA inhibits macrophage migration [45]. Consistently, our present study showed that Tan IIA attenuated LPS-induced HSC migration in both wound-healing and trans-well invasion assays.

HSCs express a multitude of chemokines, including the CC chemokines CCL2, CCL3, and CCL5, which are involved in cell chemotaxis and liver fibrosis [49], [50]. Chemokines and their receptors are up-modulated in the injured liver [51]. In line with this, we observed that Tan IIA and Sal B suppressed LPS-induced mRNA expressions of *CCL2, CCL3,* and *CCL5* genes. Notably, LPS triggered gene expression of *CCL3* to 9.3 times, in addition, Tan IIA and Sal B inhibited *CCL3* efficiently. Seki et al. and Brenner et al. both indicated that HSC is directly stimulated by TLR4 to trigger proinflammatory features, such as upregulation of CCL2, CCL3 and ICAM-1 [15], [16]. Furthermore, researchers found that in human HSC, all three MAPKs induce CCL2 secretion [34] and CCL3 stimulates cell migration [35]. There is report mentioned JNK-2 and p38 are required for LPS related RANTES (CCL5) mRNA expression [36].

A previous study showed that Tan IIA exerts anti-inflammatory effects against LPS by downregulating *iNOS* gene expression, NO production, and expressions of inflammatory cytokines (IL-1β, IL-6, and TNF-α) in macrophages [52]. TNF-α and IL-1 are reported as acute-response cytokines [53], [54], and mediate activation of NF-κB pathway in HSCs [55]. On the other hand, IL-6 directly promotes HSC survival and proliferation during enhanced liver fibrosis [56]. Our present study showed that Tan IIA inhibited LPS-stimulated inflammatory response, reducing mRNA expressions of *IL-1β, TNF-α, IL-6,* and *iNOS* genes, which are NF-κB dependent. In our previous paper, Sal B reduced ROS accumulation in primary rat hepatocytes and HSCs, and diminished α-SMA formation in HSCs [23]. In the present study, we found that both Tan IIA and Sal B inhibited mRNA expression of *α-SMA* gene, with Tan IIA exerting more powerful effects. Tan IIA exerted inhibited LPS-induced NF-κB activation and translocation, downregulated MAPKs phosphorylations as well, together with attenuating chemotaxis and mRNA expressions of *CCL2, CCL3,* and *CCL5* genes in HSCs. Tan IIA also suppressed mRNA expressions of *α-SMA* and *IL-1β, TNF-α, IL-6,* and *iNOS* genes. In conclusion, our results suggest that Tan IIA inhibited LPS- induced HSC activation, which might hold potential for treating hepatic fibrosis.

## Supporting Information

Figure S1 There was no proliferation in HSC-T6 cells with Tan IIA and Sal B treatment. HSC-T6 cells were seeded on glass cover slips in 6 well-plates (1×10^6^ cells/well) and pretreated with Tan IIA (10 µM) or Sal B (200 µM) for 1 hr, then exposed to LPS for 24 hr. The protocols were followed according to the method described previously. The primary antibody was against Ki67 (1∶100, Abcam, Cambridge, UK). The cells were visualized on LSM780 confocal microscope (Zeiss, Oberkochen, Germany) using a digital imaging system. We took photos for ten fields randomly of each group. The results show that there was no increase in proliferation by Tan IIA or Sal B.
(TIF)

Figure S2 Pyrrolidine dithiocarbamate (PDTC) inhibited NF-κB luciferase activities in HSC-T6 cells. We used NF-κB inhibitor- pyrrolidine dithiocarbamate (PDTC, 1, 5, 10 and 25 µM) for comparison in the Luciferase assay. Samples were harvested according to the methods described previously (n = 3). PDTC reduced NF-κB activities significantly at 10 and 25 µM. We measured five downstream genes of NF-κB by real-time PCR. We pretreated HSC-T6 cells with PDTC (25 µM) for 1 hr, then exposed to LPS (100 ng/ml). Samples were harvested according to the methods described previously (n = 3). PDTC at 25 µM exerted significant inhibition of LPS-stimulated mRNA expressions of *IL-1β, TNF-α, iNOS, ICAM-1* and *IL-6* genes, respectively.
(TIF)

Table S1 Gene sequences of forward and reverse primers. Quantitative real-time PCR analysis for the expressions of chemokine (C-C motif) ligand 2 (CCL2), CCL3, CCL5, interleukin-1β (IL-1β), tumor necrosis factor-α (TNF-α), interleukin-6 (IL-6), intercellular adhesion molecule-1 (ICAM-1), iNOS, α-smooth muscle actin (α-SMA), GAPDH.
(TIF)
