# Supplementary figures and images for: Inhibitory Effect of Tanshinone IIA on Rat Hepatic Stellate Cells
Source: PLoS One. 2014 Jul 30;9(7):e103229. doi: 10.1371/journal.pone.0103229 (PMC4116159; doi:10.1371/journal.pone.0103229)

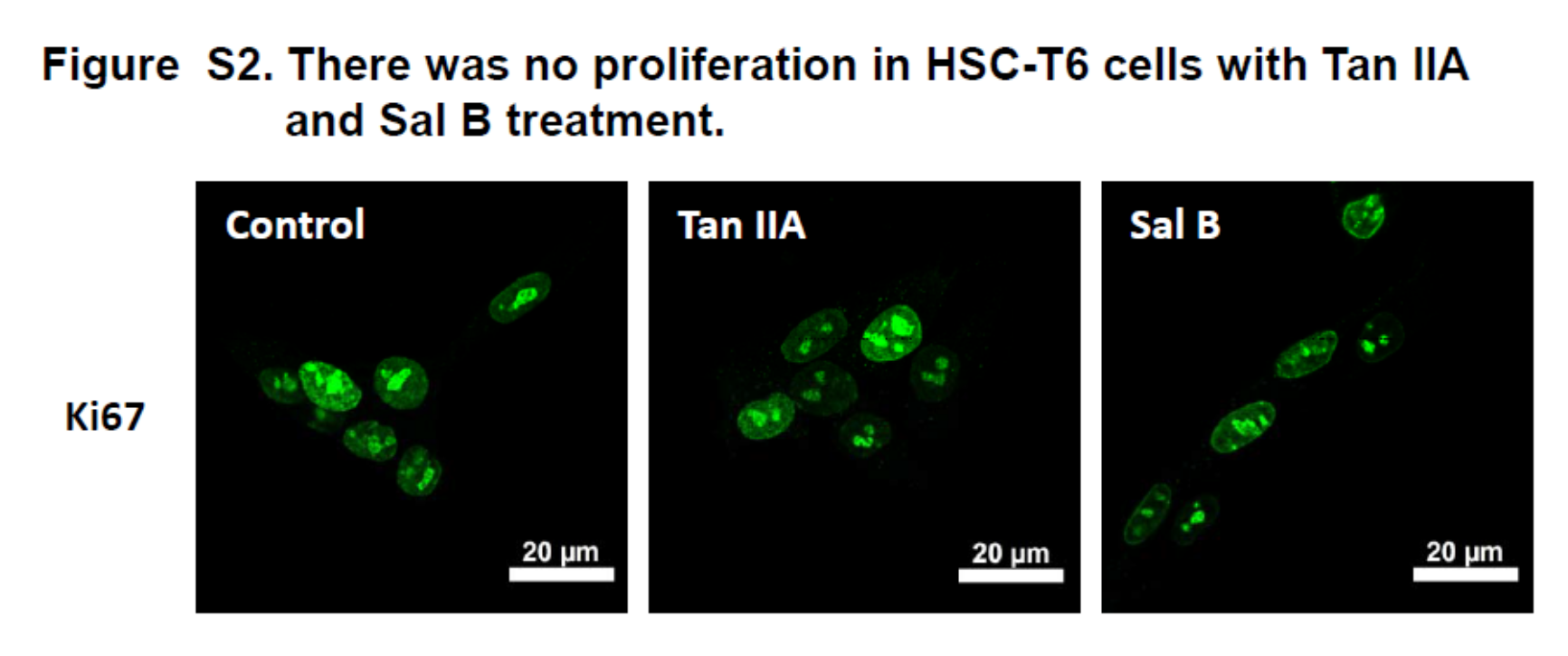

Supplement: Figure S1 — There was no proliferation in HSC-T6 cells with Tan IIA and Sal B treatment. HSC-T6 cells were seeded on glass cover slips in 6 well-plates (1×10^6^ cells/well) and pretreated with Tan IIA (10 µM) or Sal B (200 µM) for 1 hr, then exposed to LPS for 24 hr. The protocols were followed according to the method described previously. The primary antibody was against Ki67 (1∶100, Abcam, Cambridge, UK). The cells were visualized on LSM780 confocal microscope (Zeiss, Oberkochen, Germany) using a digital imaging system. We took photos for ten fields randomly of each group. The results show that there was no increase in proliferation by Tan IIA or Sal B. (TIF) [file pone.0103229.s001.tif]

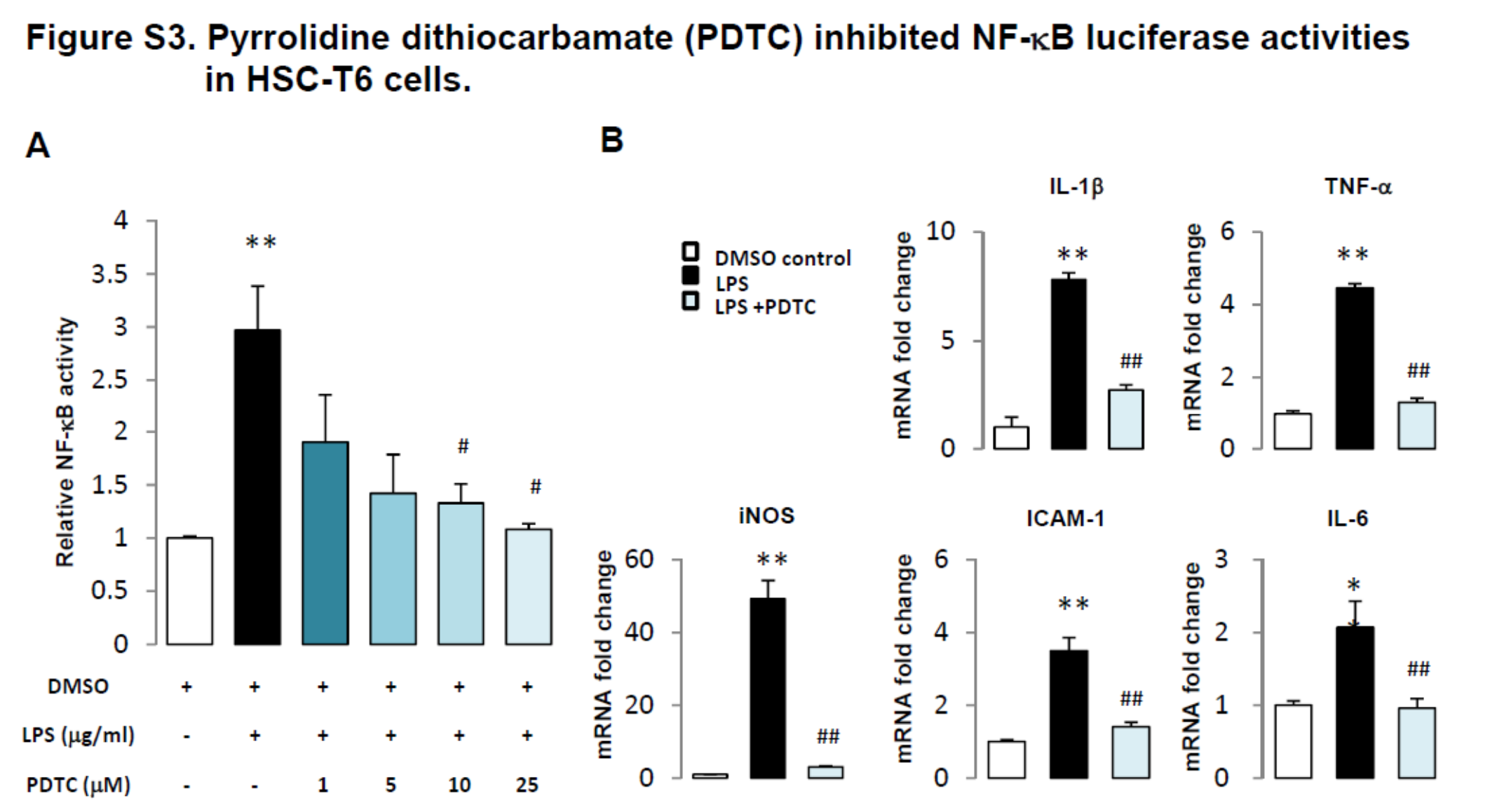

Supplement: Figure S2 — Pyrrolidine dithiocarbamate (PDTC) inhibited NF-κB luciferase activities in HSC-T6 cells. We used NF-κB inhibitor- pyrrolidine dithiocarbamate (PDTC, 1, 5, 10 and 25 µM) for comparison in the Luciferase assay. Samples were harvested according to the methods described previously (n = 3). PDTC reduced NF-κB activities significantly at 10 and 25 µM. We measured five downstream genes of NF-κB by real-time PCR. We pretreated HSC-T6 cells with PDTC (25 µM) for 1 hr, then exposed to LPS (100 ng/ml). Samples were harvested according to the methods described previously (n = 3). PDTC at 25 µM exerted significant inhibition of LPS-stimulated mRNA expressions of IL-1β, TNF-α, iNOS, ICAM-1 and IL-6 genes, respectively. (TIF) [file pone.0103229.s002.tif]

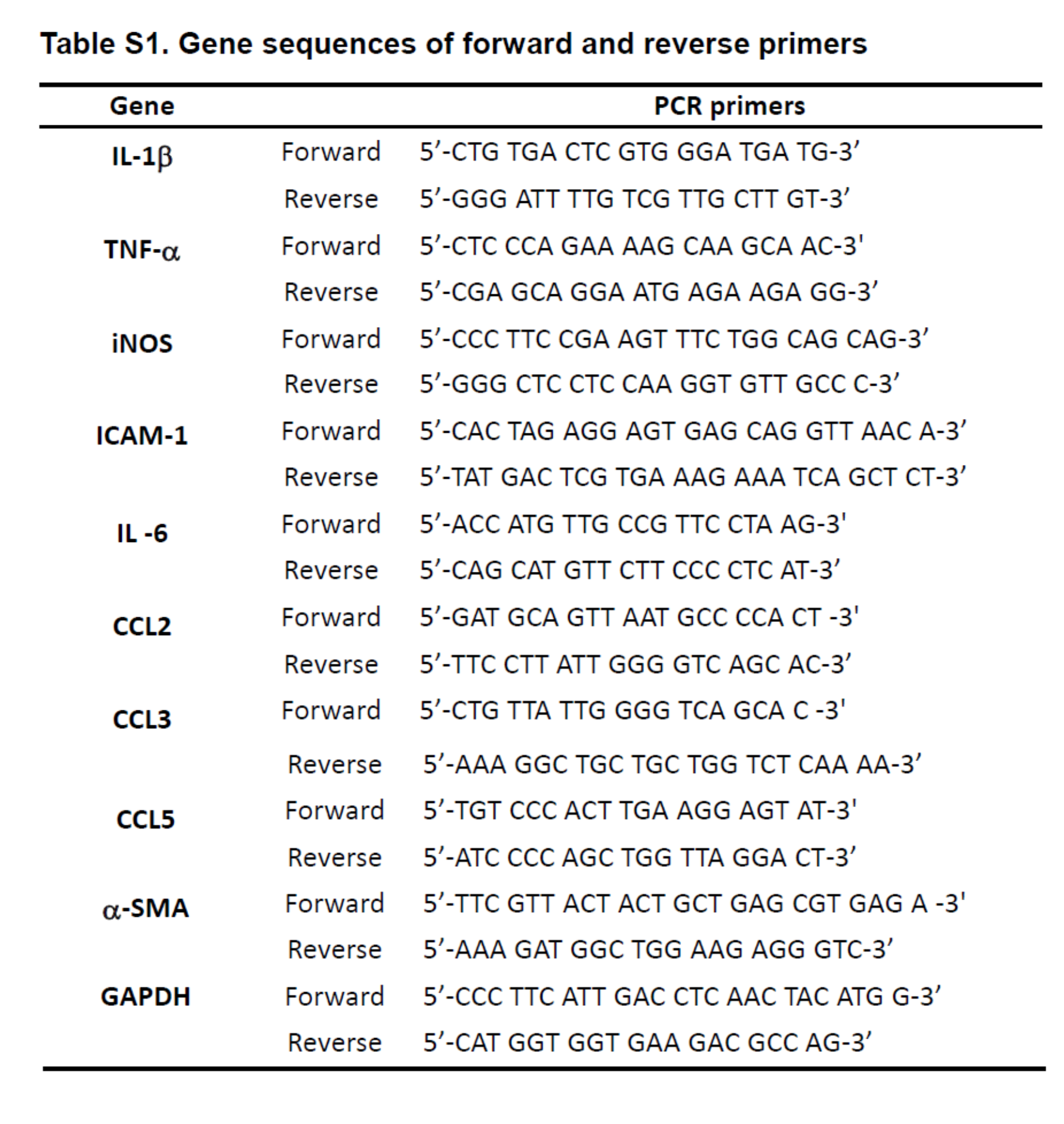

Supplement: Table S1 — Gene sequences of forward and reverse primers. Quantitative real-time PCR analysis for the expressions of chemokine (C-C motif) ligand 2 (CCL2), CCL3, CCL5, interleukin-1β (IL-1β), tumor necrosis factor-α (TNF-α), interleukin-6 (IL-6), intercellular adhesion molecule-1 (ICAM-1), iNOS, α-smooth muscle actin (α-SMA), GAPDH. (TIF) [file pone.0103229.s003.tif]
